# Fouling Mitigation of Silicon Carbide Membranes by Pre-Deposited Dynamic Membranes for the Separation of Oil-in-Water Emulsions

**DOI:** 10.3390/membranes15070195

**Published:** 2025-06-30

**Authors:** Xin Wu, Minfeng Fang, Guanghui Li

**Affiliations:** 1 Innovation Centre for Environment and Resources, School of Chemistry and Chemical Engineering, Shanghai University of Engineering Science, 333 Longteng Road, Shanghai 201620, Chinaghli@sues.edu.cn (G.L.); 2 China Petroleum and Chemical Industry Key Laboratory of Silicon Carbide Ceramic Membrane, Shanghai University of Engineering Science, 333 Longteng Road, Shanghai 201620, China; 3 Zhejiang Motonghuihai Sci & Tech Development Co., Ltd., 1558 Dongpo Road, Huzhou 313000, China

**Keywords:** membrane fouling, fouling mitigation, silicon carbide membranes, dynamic membranes, pre-deposited DM, oil-in-water emulsion, parameter optimization

## Abstract

Membrane fouling poses a significant challenge in the widespread adoption and cost-effective operation of membrane technology. Among different strategies to mitigate fouling, dynamic membrane (DM) technology has emerged as a promising one for effective control and mitigation of membrane fouling. Silicon carbide (SiC) membranes have attracted considerable attention as membrane materials due to their remarkable advantages, yet membrane fouling is still inevitable in challenging separation tasks, such as oil-in-water (O/W) emulsion separation, and thus effective mitigation of membrane fouling is essential to maximize their economic viability. This study investigates the use of pre-deposited oxide DMs to mitigate the fouling of SiC membranes during the separation of O/W emulsions. Among five screened oxides (Fe_2_O_3_, SiO_2_, TiO_2_, ZrO_2_, Al_2_O_3_), SiO_2_ emerged as the most effective DM material due to its favorable combination of particle size, negative surface charge, hydrophilicity, and underwater oleophobicity, leading to minimized oil droplet adhesion via electrostatic repulsion to DM surfaces and enhanced antifouling performance. Parameter optimization in dead-end mode revealed a DM deposition amount of 300 g/m^2^, a transmembrane pressure (*TMP*) of 0.25 bar, and a backwashing pressure of 2 bar as ideal conditions, achieving stable oil rejection (~93%) and high pure water flux recovery ratios (*FRR*, >90%). Cross-flow filtration outperformed dead-end mode, maintaining normalized permeate fluxes of ~0.4–0.5 (cf. ~0.2 in dead-end) and slower *FRR* decline, attributed to reduced concentration polarization and enhanced DM stability under tangential flow. Optimal cross-flow conditions included a DM preparation time of 20 min, a *TMP* of 0.25 bar, and a flow velocity of 0.34 m/s. The results establish SiO_2_-based DMs as a cost-effective strategy to enhance SiC membrane longevity and efficiency in O/W emulsion separation.

## 1. Introduction

Membrane technology has emerged as a vital tool in the separation and purification of complex mixtures, with widespread applications across water treatment, food processing, and the petrochemical industry [1,2,3,4]. Membranes offer several advantages, including high separation efficiency, modularity, compact design, and scalability. Despite the advantages, one of the primary limitations of membrane technology is membrane fouling, a phenomenon characterized by the accumulation of particles, colloids, or biological matter on the membrane’s surface or within its pores [5,6,7]. Fouling leads to a decline in flux, increased transmembrane pressure (*TMP*), and eventual loss of membrane performance, thereby raising operational costs and shortening membrane lifespan. Effective control and mitigation of fouling are therefore critical for the performance and economic feasibility of membranes.

Among the diverse range of membrane materials available, silicon carbide (SiC) membranes have attracted considerable attention due to their remarkable chemical stability, high mechanical strength, exceptional hydrophilicity, and excellent thermal resistance [8,9,10]. These features allow SiC membranes to operate under challenging conditions, making them especially suitable for robust separation tasks. SiC membranes are generally manufactured through high-temperature recrystallization of raw SiC powders, which involves costly raw materials and energy-intensive sintering processes. Given the attractive advantages of SiC membranes and the high cost of their fabrication, effective fouling mitigation strategies are essential to maximize their economic viability. Multiple strategies have been developed to address membrane fouling, including physical cleaning (e.g., backwashing, air scouring), chemical cleaning, surface modification, and the application of anti-fouling coatings [11,12,13,14,15,16,17,18]. However, these methods often involve significant maintenance demands, increased chemical usage, or complex manufacturing processes.

Dynamic membrane (DM) technology has emerged as a promising membrane fouling mitigation strategy [19,20,21,22,23]. A DM is a cake layer or secondary membrane that is pre-deposited or self-forms in situ on a primary membrane (PM) surface. This layer acts as a renewable and sacrificial filtration barrier, capturing foulants before they can reach and impair the underlying PM. By localizing fouling to the easily removable DM layer, the PM remains protected, facilitating longer operational cycles and simplified cleaning protocols. In the context of SiC membranes, integrating DM technology presents an alternative approach to harnessing the inherent durability of SiC while effectively minimizing the adverse impacts of fouling.

Among the diverse applications of membrane technology, the separation of oil-in-water (O/W) emulsions presents a particularly complex and significant challenge due to the stability of emulsions and the associated fouling issues [24,25]. O/W emulsions are heterogeneous mixtures of oil droplets dispersed in water stabilized by surfactants, polymers, or fine particles. These emulsions are commonly generated in industries like petrochemicals, food processing, and wastewater treatment. Their separation is critical to meet stringent environmental regulations and recover valuable resources. However, the stable nature of emulsions—characterized by small droplet sizes and high interfacial tension—poses a significant challenge for effective separation. Conventional methods, such as gravity separation and chemical treatments, are often insufficient, necessitating advanced separation technologies like membrane filtration, but the fouling issues have to be effectively mitigated.

In this study, we explore the DM strategy to mitigate the fouling of SiC membranes during the separation of O/W emulsions. A wide variety of DM materials have been used in the literature, among which oxides are the most commonly studied materials for the formation of DMs. While different individual studies have explored specific oxides, there is a lack of comparative studies of different oxides for the formation of DMs and their fouling mitigation performance in separation. We aim to prepare pre-deposited DMs using five oxides (Fe_2_O_3_, SiO_2_, TiO_2_, ZrO_2_, Al_2_O_3_) and study their properties (surface charges, surface roughness, hydrophilicity, and oleophobicity) as well as their performance in the separation of O/W emulsions, thereby identifying the most suitable DM material for our study. Then, based on this type of DM, we systematically study the effects of different operational parameters on its performance during O/W emulsion separation in dead-end and cross-flow mode, respectively, with an emphasis on fouling mitigation for SiC membranes to understand the ruling parameters for fouling control and to optimize operational parameters to enhance fouling resistance in different modes. Finally, we compare DM performance in two modes to obtain insights into the most effective design and implementation of DM systems for the separation of O/W emulsions.

## 2. Experimental

### 2.1. Materials

Flat-sheet SiC microfiltration membranes (average pore size: 0.4 µm; porosity: 40%) were supplied by Zhejiang Motonghuihai Sci & Tech Development Co., Ltd. (Huzhou, China). Oxide particles of Fe_2_O_3_, SiO_2_, TiO_2_, ZrO_2_, and Al_2_O_3_ (purity: >99.99%; nominal average size: 1 µm for all) were provided by Guangzhou Metallurgical Group Co., China. Vegetable oil, Tween 80 (C_64_H_124_O_26_, AR), sodium hydroxide (NaOH, 98%), hydrochloric acid (HCl, AR), and tetrachloroethylene (C_2_Cl_4_, AR) were purchased from Shanghai Titan Technology Co.(Shanghai, China). Distilled water (Watson’s, Guangzhou, China, conductivity < 2 µS/cm) was employed in all experiments.

### 2.2. Preparation of O/W Emulsions and DM Particle Suspensions

The O/W emulsions (2000 mg·L^−1^) were prepared using vegetable oil and Tween 80 as the base oil and the surfactant, respectively. Vegetable oil and Tween 80 in a mass ratio of 1:1 were mixed in distilled water, and the mixture was homogenized using a high-shear homogenizing emulsifier (HR-500D, HUXI, Shanghai, China) at 12,000 rpm for 5 min. The concentration of O/W emulsions in most membrane separation studies typically ranges from 100 to 1000 ppm to simulate industrial or municipal wastewater [26,27,28,29,30], though a few exceed 1000 ppm [31,32]. We selected a concentration of 2000 ppm for our study, balancing emulsion stability, experimental control, and a more effective demonstration of DM performance.

The suspensions of DM particles (1 g·L^−1^) were prepared using different oxide materials in distilled water. A specific type of oxide particle was weighed to 1.0 g and mixed into 1.0 L of distilled water, followed by thorough homogenization with a magnetic stirrer (84–1A6S, SILE, Shanghai, China) at 800 rpm for 10 min. Both O/W emulsions and DM particle suspensions remain stable for an extended period.

### 2.3. DM Filtration of O/W Emulsions in Dead-End Mode

**Figure 1:**
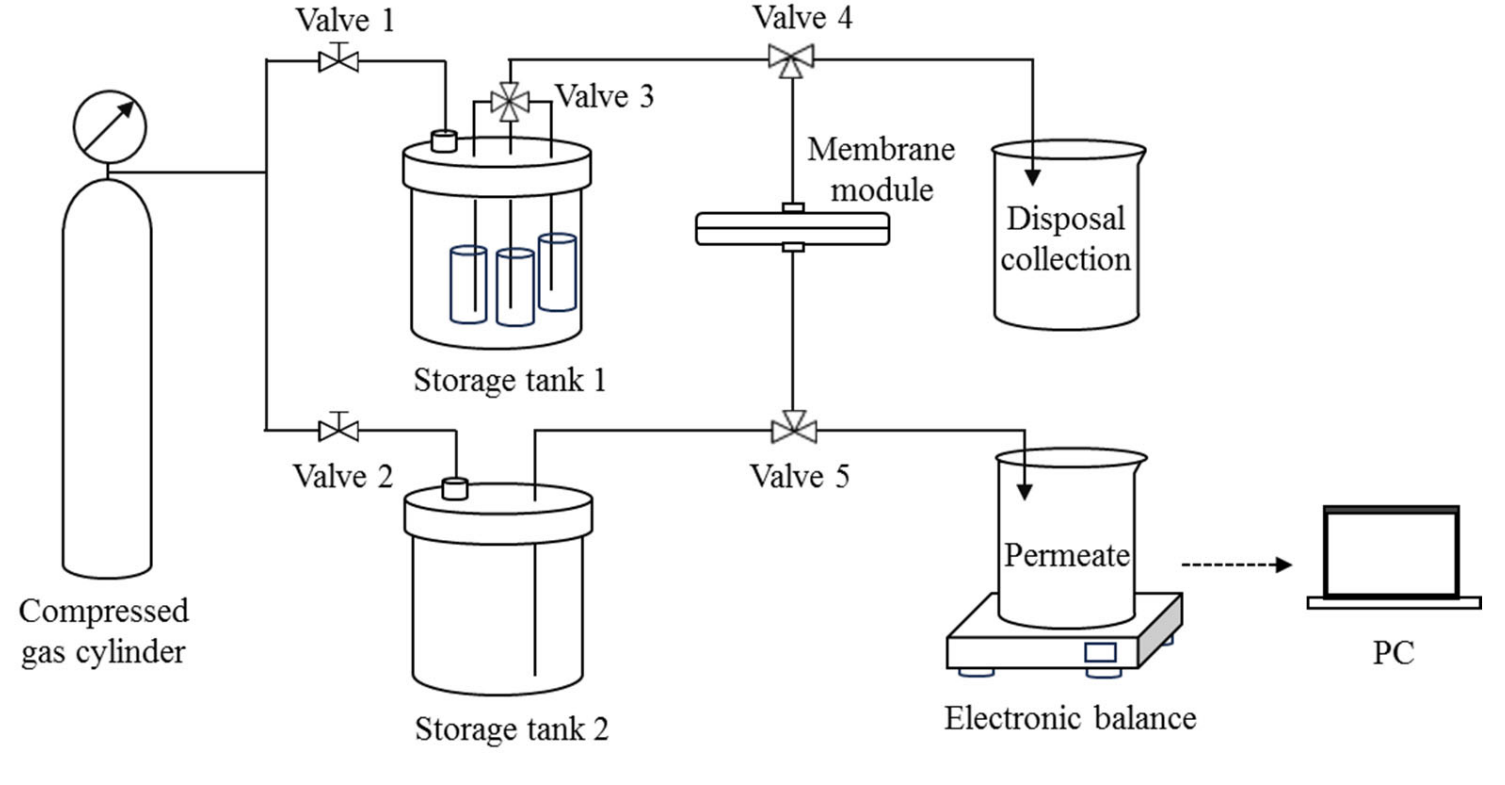
Schematic diagram of the dead-end filtration setup with backwash function.

A dead-end filtration setup with feed switching and backwashing functions was specifically designed for the experiment (Figure 1). The membrane module holds a disk-shaped SiC membrane with an effective filtration area of 3.14 cm^2^. Storage tank 1 holds three separate containers with a suspension of DM material, an O/W emulsion, and distilled water, all of which are connected to a multi-port valve (Valve 3). Storage tank 2 contains distilled water for backwashing. The *TMP* during filtration and backwashing is provided by the compressed gas from the cylinder via Valve 1 and Valve 2. Throughout the filtration process, the permeate mass is measured in real time by an electronic balance connected to a PC, and a curve of permeate mass vs. time can be recorded.

The DM filtration of O/W emulsions consists of repeating cycles of four steps: pure water flux (*PWF*) measurement, DM formation, filtration of O/W emulsion, and DM removal. The details of each step are outlined below.

#### 2.3.1. *PWF* Measurement

Distilled water in Storage tank 1 is allowed to pass through the membrane module by setting Valves 3, 4, and 5 correctly. Filtration continues for 1 min, and the change in permeate mass is automatically recorded in real time, from which the *PWF* can be deduced.

#### 2.3.2. DM Formation

With a specified *TMP*, Valve 3 is set to allow the suspension of DM material in Storage tank 1 to flow out and form a DM on the surface of the SiC membrane. Filtration is stopped once a specified volume of permeate is collected and the amount of deposited oxide particles on the SiC membrane surface can be calculated. The DM deposition amount is expressed as mass per unit area, measured in g/m^2^.

#### 2.3.3. Filtration of O/W Emulsions

The O/W emulsion is then directed through the newly formed DM by adjusting Valve 3 to maintain a specific *TMP* for 30 min, until the flux drops below 20% of its original value.

#### 2.3.4. DM Removal

The flow direction through the DM and the PM is then reversed by setting Valve 4 and 5 accordingly, and the distilled water in Storage tank 2 is allowed to backflush the DM into the disposal container. The backwashing process lasts for a certain amount of time under a *TMP* of 2 bar.

### 2.4. DM Filtration of O/W Emulsions in Cross-Flow Mode

**Figure 2:**
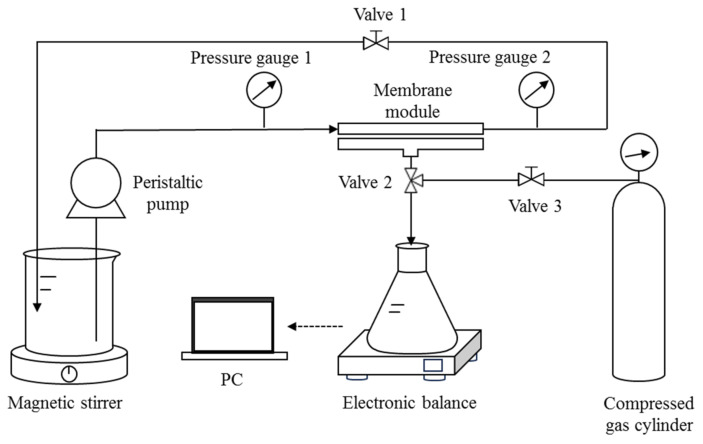
Schematic diagram of the cross-flow filtration setup with backwash function.

The cross-flow filtration setup consists of a 15 cm long and 5 cm wide membrane module with an effective filtration area of 25.07 cm^2^ (Figure 2) connected to a compress gas cylinder for backwashing. The flow rate and the *TMP* can be controlled by the peristaltic pump and Valve 1, respectively. The operating steps of each cycle of cross-flow filtration are similar to those of the aforementioned dead-end mode, except that the feed (distilled water, DM solid suspensions, or O/W emulsions) is manually changed in each step. Backwashing is achieved by allowing pressurized gas to flush from the opposite side of the membrane while maintaining the circulation of distilled water.

### 2.5. Evaluation of DM Filtration Performance

The DM filtration performance for O/W emulsions is primarily evaluated based on the permeate flux (*J*), the pure water flux recovery ratio (*FRR*), and the oil rejection rate (*R*).

*J* is calculated as follows:

$$J=\frac{V}{At}$$

where *V* (L) is the permeate volume during the filtration of O/W emulsions or distilled water, *A* (m^2^) denotes the membrane area, and *t* (h) represents filtration duration.

*FRR* is used to evaluate the fouling resistance of a membrane and is calculated as follows:

$$FRR=\frac{J_{n}}{J_{0}}\times 100\%$$

where *J_n_* (L·m^−2^·h^−1^) and *J*_0_ (L·m^−2^·h^−1^) indicate the *PWF* after each backwash (*n* = 1, 2, 3, 4) and the initial *PWF* of the SiC membrane, respectively.

*R* is calculated using the following formula:

$$R=\frac{C_{f}-C_{p}}{C_{f}}\times 100\%$$

where *C*_f_ (mg·L^−1^) and *C*_p_ (mg·L^−1^) are the oil concentrations of the feed and the permeate, respectively.

### 2.6. Analytical Methods

The oil concentration in O/W emulsions and the permeate was quantified using an FTIR spectrometer (Nicolet iS20, Thermo Fisher Scientific Inc., Waltham, MA, USA). The size distribution and zeta potentials of the oxide particles in suspension and oil droplets in O/W emulsions were measured using a particle size and zeta potential analyzer (Nano ZS90, Malvern Panalytical, Malvern, UK). The surface morphology of the membrane was observed using a white light interference laser microscope (VK-X3050, Keyence Corp., Osaka, Japan), and the surface roughness was subsequently analyzed. The water contact angle (*WCA*) of the membrane in air and the underwater oil contact angle (*OCA*) were measured using a drop shaper analyzer (DSA30S, KRÜSS Scientific, Hamburg, Germany). During the measurement of the *WCA* in air, it was noted that the porous nature of the membrane makes *WCA* measurements difficult because the water droplet will penetrate rapidly into the membrane, so the change in *WCA* vs. time was recorded and plotted instead. The dynamic assessment of the *WCA* provided insights into the wettability behavior of the membranes as a function of the water penetration rate [29].

## 3. Results and Discussion

### 3.1. Properties of O/W Emulsions, DM Materials, and DMs

The properties of DM material (particle size, surface charge, etc.) and the resultant DM (surface roughness, hydrophilicity, oleophobicity, etc.) can largely determine the performance of the DM in operation. To prepare a DM with optimum performance for O/W emulsion separation, a pre-screening of DM material was first carried out. Figure S1a shows the size distribution of the different oxides investigated, as well as the oil droplets of the O/W emulsion (2000 mg·L^−1^). The size distribution for five oxides (Fe_2_O_3_, ZrO_2_, Al_2_O_3_, SiO_2_, and TiO_2_) is very similar to each other, with the peak size near 1 µm, wherein Fe_2_O_3_ and TiO_2_ have wider distribution and the distribution of SiO_2_, ZrO_2_, and Al_2_O_3_ is relatively narrower. The smallest particle size for Al_2_O_3_, SiO_2_, and TiO_2_ is about 600 nm, which is larger than the size of the majority pores of the SiC membrane as the PM (average pore size 400 nm). Thus, the DM formed by these oxides on the SiC PM can be roughly categorized as Class I type [20] and can be effectively removed by backwashing. Meanwhile, the size distribution of the oil droplets in O/W emulsions is also narrow, with the majority lying between 500 nm and 1000 nm (Figure S1a). As a result, the pores of a DM created through the stacking of the aforementioned particles can be expected to maintain a good oil rejection rate while ensuring satisfactory flux.

On the other hand, the surface charge of the DM and oil droplets also plays an important role in the DM filtration of O/W emulsions. The prepared O/W emulsions remained stable for an extended period thanks to the negative charge of oil droplets, leading to repulsion among droplets. The pH of the prepared O/W emulsions was measured to be 5.6, and the droplets had a zeta potential of −26.70 ± 0.64 mV, consistent with the reported value [28]. The zeta potentials of the particles of all five oxides in suspensions decrease with increasing pH in the range of 3 to 10 (Figure S1b). Table S1 lists the isoelectric points of the five oxides along with their zeta potentials measured at pH 5.6. The isoelectric points for Fe_2_O_3_, TiO_2_, ZrO_2_, and Al_2_O_3_ particles are 6.7, 5.7, 6.8, and 7.0, respectively (Table S1), suggesting that the surface of the DM prepared from all four materials exhibits positive charges during O/W emulsion separation (pH = 5.6) and leads to electrostatic attraction between oil droplets and the DM’s surface. In contrast, SiO_2_ particles, with an isoelectric point of 3.2, have a negative surface charge in O/W emulsions, resulting in electrostatic repulsion between the SiO_2_ DM and oil droplets. The electrostatic interaction between the membrane surface and foulants can exacerbate membrane fouling, while electrostatic repulsion can alleviate fouling [33]. Based on these, SiO_2_ is likely the most suitable DM material for O/W emulsion separation.

**Figure 3:**
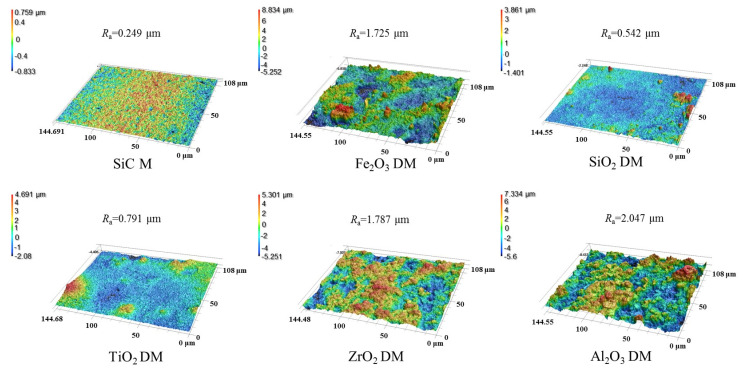
Surface roughness of SiC membrane and different oxide DMs.

**Figure 4:**
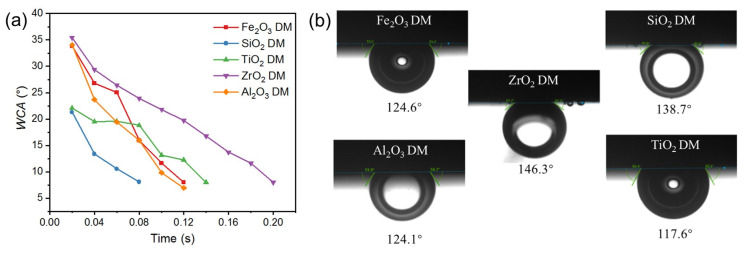
(**a**) The evolution of the *WCA* over time for different DMs. (**b**) Underwater *OCA* for different DMs.

The roughness and the wetting behavior of the surface of the DM prepared from different oxides were also investigated to evaluate the interaction of the DM surface and oil droplets in O/W emulsions. After a DM layer was deposited onto the SiC PM, the surface morphology was greatly altered, as verified by the comparison of SEM surface images for the pristine SiC PM and the Fe_2_O_3_ DM as an example (Figure S2). We mainly used 3D laser microscopy to observe surface morphology and analyze the roughness of surfaces. Figure 3 depicts the surface morphology of the SiC PM and different DMs prepared by depositing oxides in an amount of 300 g/m^2^ onto the surface. It can be seen that the surface roughness increased significantly after the formation of the DMs, with *R*_a_ values of 0.249 µm for the SiC PM and 1.72, 0.542, 0.791, 1.787, and 2.047 µm for five DMs, implying increased repulsion to the hydrophobic foulants for the DMs compared to that for the SiC PM, as the roughening of the surface can usually enhance both the hydrophilicity and fouling mitigation of membranes [34,35]. As for different DMs, their fouling mitigation ability can be distinguished by the results of *WCA* and *OCA* measurements (Figure 4). In *WCA* measurement for porous materials, the rate of water infiltration is influenced not only by the hydrophilicity but also by the porosity of the material [36]. Because water can permeate through porous DM in air quickly, plots of *WCA* vs. time are presented instead of static *WCA*s (Figure 4a), showing that the rate of *WCA* change follows an order of ZrO_2_ < TiO_2_ < Fe_2_O_3_ ≈ Al_2_O_3_ ≈ SiO_2_. Considering the similarity of the size distributions of all oxide particles, the different rates of *WCA* change are mostly due to the hydrophilicity difference. In addition, SiO_2_ and TiO_2_ have the lowest starting *WCA*s at *t* = 0.02 s among all oxides. Thus, SiO_2_ could be the ideal material for the DM in terms of hydrophilicity. Hydrophilicity is a crucial surface property of filtration media [37]. Hydrophilic surfaces effectively prevent the deposition or adsorption of hydrophobic oil droplets in emulsions [38]. Besides hydrophilicity, however, oleophobicity is more indicative of the repulsion properties of DM surfaces against oil droplets in the O/W emulsion filtration. The greater the oleophobicity, the greater the ability of the resulting DM to mitigate oil fouling. Thus, the *OCA*s of different oxide DMs were measured and compared, with the results shown in Figure 4b. It can be seen that all DM surfaces are oleophobic, with all *OCA* values above 117° and an order of *OCA* values as follows: TiO_2_ < Al_2_O_3_ ≈ Fe_2_O_3_ < SiO_2_ < ZrO_2_. SiO_2_ and ZrO_2_ DM surfaces have *OCA* values of 138.7° and 146.3°, suggesting that SiO_2_ is among the best two DM materials in terms of oleophobicity.

Based on the combined studies of particle size and surface electrical property for oxide particles and surface morphology and wettability (hydrophilicity and oleophobicity) for the resulting DMs, SiO_2_ is the most suitable DM material for O/W emulsion separation in our study.

### 3.2. Performance Comparison of Various DMs and SiC PM Itself in the Dead-End Filtration of O/W Emulsions

**Figure 5:**
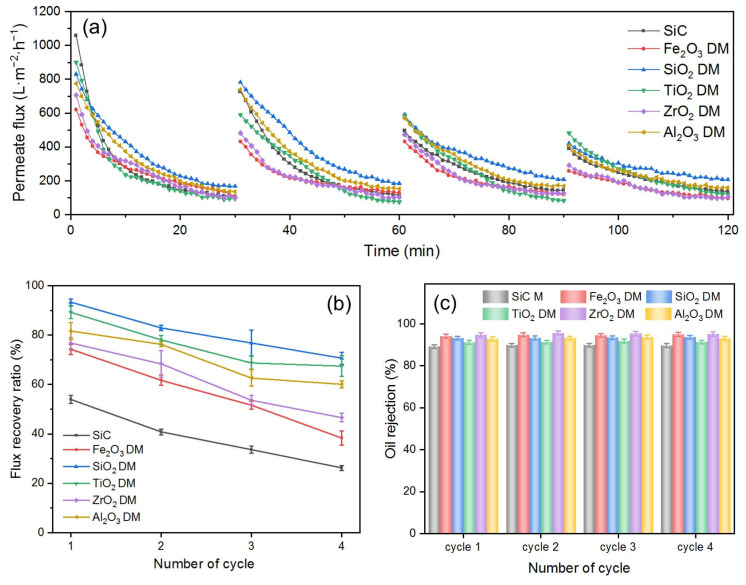
(**a**) *J*, (**b**) *FRR*, and (**c**) *R* for SiC PM and different oxide DMs when filtering O/W emulsions (*TMP*: 0.25 bar; backwashing pressure: 2 bar). The variations in the calculated flux values for repeat experiments were typically within 3%.

To assess the fouling mitigation capabilities of DMs and compare the performance of the aforementioned oxides as DM materials, a series of DMs were prepared, each consisting of oxide particles with an amount of 300 g/m^2^. Their effectiveness in O/W emulsion separation was evaluated using a dead-end filtration setup chosen for its simplicity in swiftly identifying key influencing parameters. The SiC membrane itself was also used as the control. The results are shown in Figure 5.

As depicted in Figure 5a, the *J*s of six DMs all gradually decrease over time in every filtration cycle, a phenomenon attributed to membrane fouling [39]. In the first cycle, the initial permeate flux (*J*_i_) follow an order of SiC PM > TiO_2_ DM > SiO_2_ DM > Al_2_O_3_ DM > ZrO_2_ DM > Fe_2_O_3_ DM. The absence of a DM layer on the surface of the SiC membrane resulted in the smallest flow resistance and thus the highest flux. For DMs, the *J*_i_ is influenced largely by their pore size, which is the smallest part of the voids formed by the stacking of oxide particles on the surface of the base membrane. The pore size of DMs is correlated with the size of oxide particles constituting DMs. The larger the size of the DM particles, the larger the voids formed by the stacking of the particles [40]. Among the five DM materials, TiO_2_ exhibited the largest average particle size (Table S1), resulting in the highest *J*_i_ in all DMs. Although SiO_2_ and Al_2_O_3_ have similar average particle sizes, SiO_2_ achieved a higher *J*_i_ due to its excellent hydrophilicity (Figure 4a), which is consistent with reports in the literature [41]. Although the average particle size of Fe_2_O_3_ is slightly larger than that of ZrO_2_, the *J*_i_ of Fe_2_O_3_ is lower than that of ZrO_2_. This could be explained by the wider distribution of particle size for Fe_2_O_3_ (Figure S1a), where smaller particles fill in the voids between larger particles, resulting in smaller pore sizes.

In each cycle, all *J*s declined significantly during the first 20 min before reaching a pseudo-steady state between 25 and 30 min. This pseudo-steady state is characteristic of dead-end filtration [42], and the flux at this pseudo-steady state is referred to as the stable permeate flux (*J*_s_). During all filtration cycles, the SiO_2_ DM consistently maintained the highest *J*_s_, indicating that the SiO_2_ DM exhibited the most effective fouling mitigation properties. This observation is consistent with the finding that the SiO_2_ DM has the highest *FRR* in all four cycles (Figure 5b). The *FRR* decreased progressively with the number of cycles (Figure 5b), contributing to the reduction in *J*_i_ shown in Figure 5a. This is because although DM layers are deposited on the PM and can protect the fouling of the PM from the majority of oil droplets in O/W emulsions, there is an inevitable portion of oil, such as dissolved oil or oil droplets in sub-nanometer sizes, that can still reach and foul the SiC PM. Therefore, the *J*_i_ in each cycle cannot be expected to be restored to the same value as in the previous cycle, i.e., the *FRR* in each subsequent cycle keeps decreasing. This finding aligns with previous reports in the literature [28,43,44]. The *FRR*s of all DMs were generally higher than that of the SiC membrane throughout the four cycles, suggesting that the overall fouling mitigation of the DM is superior to that of the SiC membrane alone. The *FRR*s of the five DMs rank as follows: SiO_2_ DM > TiO_2_ DM > Al_2_O_3_ DM > ZrO_2_ DM > Fe_2_O_3_ DM. This order is directly correlated to the surface properties of the DMs. A comparison between the Fe_2_O_3_ DM and the ZrO_2_ DM reveals that the ZrO_2_ DM, with a larger underwater *OCA*, exhibited a higher *FRR*, despite having similar particle sizes and surface potentials to Fe_2_O_3_. The enhanced oleophobicity effectively prevents the deposition or adsorption of hydrophobic oil droplets in emulsions, supporting the reported findings [38]. The *FRR* difference between the SiO_2_ DM and the Al_2_O_3_ DM is approximately 10%, despite their similar particle sizes. This variation can be attributed to the combined effects of oleophobicity and surface potential. At pH 5.6, the negative surface charge of the SiO_2_ DM exerts an electrostatic repulsion force against oil droplets, preventing their deposition or adsorption to the DM’s surface. In contrast, the positive surface of the Al_2_O_3_ DM creates an electrostatic attraction for oil droplets, promoting their deposition or adsorption. Additionally, the SiO_2_ DM is more oleophobic, leading to a higher *FRR*.

Figure 5c presents the *R*s of the five DMs and the SiC membrane. As the number of cycles increases, *R*s remain constant, with the DMs generally exhibiting higher *R* values than the SiC membrane. This is because the stacking of DM materials on the SiC membrane results in a reduced pore size compared to that of the SiC membrane. The *R*s of different DMs also vary, following a trend consistent with the particle size order of the DM material, indicating that the *R* of DMs is primarily influenced by their pore size [40]. For the DMs with comparable pore sizes, surface properties play a critical role in oil rejection performance. This suggests that stronger electrostatic repulsion enhances underwater oleophobic properties, thereby improving oil rejection efficiency [45,46].

**Figure 6:**
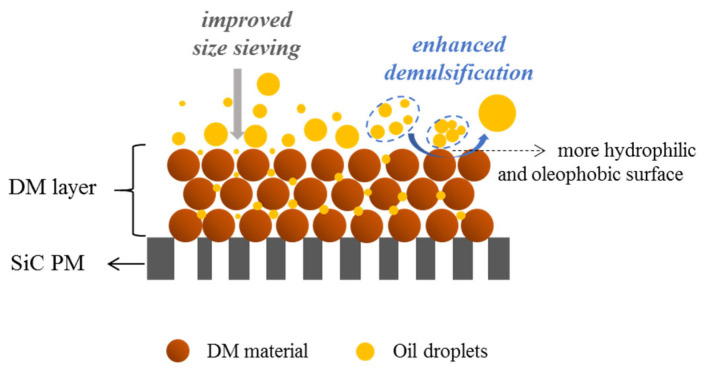
Schematic illustration of the oil rejection mechanism in DM filtration of O/W emulsions.

Regarding the oil rejection mechanism during the O/W emulsion filtration, membranes can typically achieve separation not only through the physical sieving of oil droplets larger than the membrane pores but also via demulsification. Demulsification may occur on or near the membrane’s surface due to several factors, including surfactant–surface interactions, concentration polarization, which increases the likelihood of droplet–droplet collisions, and hydrodynamic conditions that promote droplet deformation and coalescence. These mechanisms often act simultaneously to enhance oil rejection during separation. When a DM layer is deposited on the SiC PM, the improved oil rejection and O/W separation performance can be attributed to enhanced size-exclusion and demulsification effects, as illustrated in Figure 6. The stacking of DM particles reduces pore sizes, strengthening the sieving of oil droplets, while the enhanced surface wettability—specifically, increased hydrophilicity and oleophobicity—promotes demulsification. Hydrophilic surfaces attract water, accelerating oil droplet coalescence, whereas oleophobic surfaces repel oil, facilitating its separation. Among these, the improved wettability of the DM plays a more dominant role in promoting demulsification, making this mechanism a key contributor to the overall oil rejection efficiency.

In summary, the results from the performance comparison studies in DM filtration of O/W emulsions indicate that SiO_2_ is the most effective DM material for O/W emulsion separation, in accordance with the results from the properties studies of DM materials and DM surfaces in Section 3.1.

### 3.3. Influence of Operating Parameters on DM Performance During the Dead-End Filtration of O/W Emulsions

In order to investigate the effect of operating parameters (i.e., DM deposition amount, *TMP*, and backwashing pressure) on the filtration performance in dead-end filtration mode, SiO_2_ was selected as the DM material for O/W separation according to the discussion above.

#### 3.3.1. Effect of DM Deposition Amount

**Figure 7:**
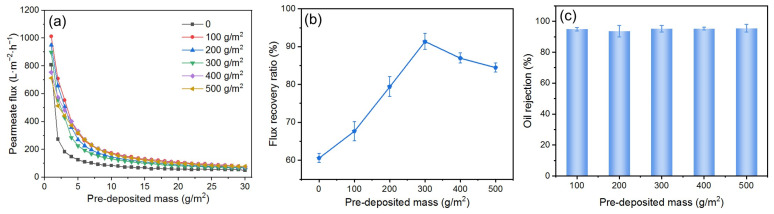
(**a**) *J*, (**b**) *FRR*, and (**c**) *R* for DM filtration of O/W emulsions with different DM deposition amounts (DM material: SiO_2_; *TMP*: 0.25 bar; backwashing pressure: 2 bar).

Because the membrane area for filtration in this study is kept constant, the deposition amount of DM material during DM formation directly determines the thickness of the DM layer, which regulates its filtration behavior and affects its ability to mitigate fouling. To investigate the influence of the DM deposition amount on fouling mitigation, DMs with varying deposition amounts were prepared for the O/W emulsion filtration under a *TMP* of 0.25 bar and a backwashing pressure of 2 bar. The results are presented in Figure 7, where the deposition amount is expressed as mass per square meter. The *J*s of all DMs with different deposited amounts of SiO_2_ are generally higher than those of the SiC membrane; it is difficult to find a correlation between the *J*_s_ and the deposition amount (Figure 7a). However, a volcano relationship between the *FFR* and the deposition amount is evident (Figure 7b), with a peak value at 300 g/m^2^. When the deposited amount is less than 300 g/m^2^, the oil droplets may not be fully retained by the DM layer, with some droplets penetrating the DM layer and reaching the SiC PM, resulting in partially irreversible fouling of the PM. Thus, the gradual increase of the DM deposition amount allows oil droplets to be increasingly retained by the DM during O/W filtration and subsequently removed with the DM during backwashing, recovering the flux with lesser fouling of the PM. However, when the deposition amount surpasses 300 g/m^2^, it is possible that the DM layer is too thick, and it becomes difficult to fully remove during backwashing, leading to a reduction in *FRR*. For all deposition amounts, the *R* values remain around 93%, without apparent differences (Figure 7c). Therefore, a DM deposition amount of 300 g/m^2^ is considered optimum during our studies.

#### 3.3.2. Effect of *TMP*

**Figure 8:**
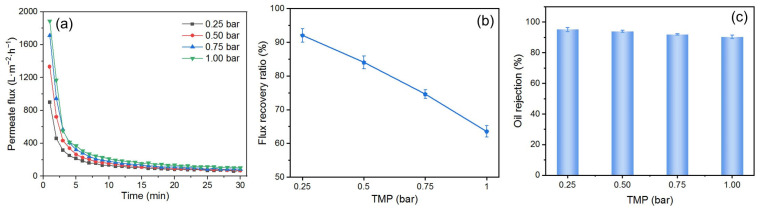
(**a**) *J* vs. time, (**b**) *FRR* vs. *TMP*, and (**c**) *R* vs. *TMP* for DM filtration of O/W emulsions with different *TMP* (DM material: SiO_2_; DM deposition amount: 300 g/m^2^; backwashing pressure: 2 bar).

The selection of *TMP* during the O/W emulsion filtration is of great importance. Due to the compressibility of oil droplets, excessively high *TMP* can cause them to compress and pass through the DM layer, leading to the fouling of the PM [45]. To investigate the effect of *TMP* on the fouling mitigation performance of the DM, experiments were conducted by preparing a DM with a deposition amount of 300 g/m^2^ and filtering the O/W emulsion at different *TMP*s, followed by backwashing at 2 bar. The results are shown in Figure 8. The *J*_i_ increases with higher *TMP*s in the range of 0.25 to 1.00 bar, and the *J*_s_ is positively correlated with the *TMP* (Figure 8a). However, at higher *TMP*s, the *J* declines more quickly, suggesting that oil droplets are more rapidly deposited on the DM’s surface, as expected. The *FRR* decreases as the *TMP* increases (Figure 8b), which can be attributed to two main factors. Firstly, the pressure exerted on the DM’s surface during filtration compresses the DM layer into the PM, making it more difficult to fully remove during backwashing, thereby reducing the *FRR*. Secondly, the compressibility of oil droplets causes them to deform under high *TMP*, allowing some of them to pass through the DM layer and reach the SiC PM, thereby contributing to PM fouling and further reducing the *FRR*. The compressibility of oil droplets also affects the *R*. There is a decrease of *R* when the *TMP* is increased from 0.25 to 1 bar (Figure 8c). At high *TMP*s, the droplets can pass through both the DM and the PM layers, leading to a reduced *R* [47]. Based on the analysis above, a *TMP* of 0.25 bar is considered to be optimum during our studies.

#### 3.3.3. Effect of Backwashing Pressure

**Figure 9:**
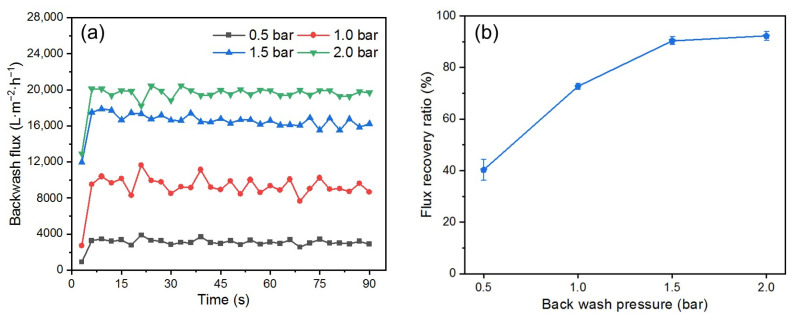
(**a**) Backwash flux, (**b**) *FRR* at different backwash pressures (DM material: SiO_2_; DM deposition amount: 300 g/m^2^; *TMP*: 0.25 bar).

One of the advantages of the DM is that its removal and cleaning process requires only a simple hydraulic backwash [17]. To determine the optimal backwashing time, flux changes during backwashing at different pressures were recorded (Figure 9a). The trend in flux was consistent for different pressures; during the first 5 s, the flux increased suddenly as the DM layer was quickly flushed away from the PM’s surface; after 5 s, the flux stabilized, indicating that the optimal cleaning state had been reached. Extending the backwash time cannot improve stable flux, so a backwash time of 10 s is enough to ensure effective cleaning.

Figure 9b shows the effect of different backwash pressures on the *FRR* after a backwash time of 10 s. The *FRR* increases with increased backwash pressure from 0.5 to 2.0 bar. However, this trend of increase begins to level off, which aligns with the findings by others [48]. When the pressure is increased from 0.5 to 1.0 bar, the *FRR* increases by approximately 30%. In contrast, increasing the pressure from 1.5 to 2.0 bar results in an *FRR* increase of less than 10%, indicating a gradual decline in backwash efficiency. It is noteworthy that at 1.5 bar, the *FRR* exceeded 90%. According to published reports [49,50], more complex membrane cleaning procedures are typically required to achieve an *FRR* of over 90%.

### 3.4. Influence of Operating Parameters on DM Performance During the Cross-Flow Filtration of O/W Emulsions

Despite the simplicity of dead-end filtration for quick analysis of the governing parameters during DM filtration and its energy efficiency, cross-flow filtration mode is more advantageous for the filtration of O/W emulsions due to its better control of concentration polarization, ability to handle high oil concentrations, and suitability for continuous operation. Therefore, the effects of operating parameters on DM filtration performance in cross-flow filtration mode were also investigated using SiO_2_ as the DM material.

#### 3.4.1. Effect of DM Preparation Time

The DM deposition amount on the surface of the PM also affects the fouling mitigation performance of the DM in cross-flow filtration. However, unlike dead-end filtration, the deposited amount of DM materials cannot be conveniently calculated using the permeate volume at a certain time. During DM preparation, the characteristics of cross-flow filtration influence particle deposition. The flow of DM particle suspensions perpendicular to the DM surface deposits DM particles onto the PM’s surface, while the parallel flow carries away some of the deposited particles. These two processes will eventually reach a stable state, resulting in a net zero change in the deposited amount of DM material. During the separation of the O/W emulsion, if the *TMP* and the flow velocity are kept the same, there is presumably no loss of DM material. Therefore, it is reasonable to use the time of the circulation of DM suspensions during DM preparation (i.e., the DM preparation time) as the varying and indicative parameter of its deposition amount. We formed the DM at a *TMP* of 0.25 bar and a flow velocity of 0.34 m/s and studied the impact of the DM preparation time on the fouling mitigation performance of the DM during the filtration of the O/W emulsion, which was also performed at a *TMP* of 0.25 bar and a flow velocity of 0.34 m/s.

**Figure 10:**
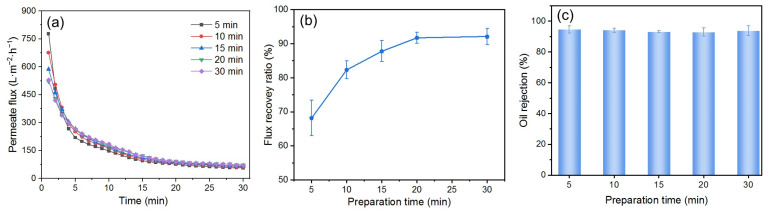
(**a**) *J*, (**b**) *FRR*, and (**c**) *R* for DM filtration of O/W emulsions with different preparation times (DM material: SiO_2_; *TMP*: 0.25 bar; flow velocity: 0.34 m/s; backwashing pressure: 2 bar).

As shown in Figure 10a, with an increased DM preparation time from 5 to 30 min, the *J*_i_ in the filtration of the O/W emulsions decreased accordingly. At a preparation time of 20 and 30 min, both the *J*_i_ and the *J*_s_ in O/W emulsion filtration seem identical. The change in the *FRR* is similar, with a gradual increase first and then a stable value (Figure 10b). This trend could be explained as follows. In the beginning, the increased preparation time leads to a thicker DM layer, which adds higher filtration resistance and results in a continuous decrease in *J*. The increased thickness also prevents oil droplets from passing through the DM layer, leading to a gradual increase in *FRR*. However, unlike dead-end filtration, the DM layer has a critical thickness in cross-flow mode for a given set of parameters beyond which the newly formed DM layer may be carried away by the fluid flow parallel to the membrane’s surface [51]. The critical thickness of the DM in this experiment seems to correspond to the preparation time of 20 min. As a result, the *J*_i_ and the *J*_s_, as well as the *FRR*, no longer increased after a preparation time of 20 min. For all preparation times, all *R*s are about 94%, essentially independent of the DM preparation time (Figure 10c). This is because the *R* is primarily determined by the pore size of the DM, which shows minimal variation across different DM preparation times.

#### 3.4.2. Effect of Flow Velocity

**Figure 11:**
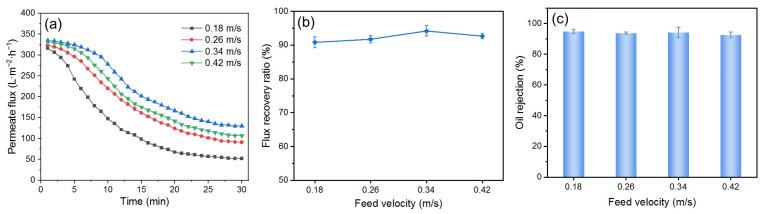
(**a**) *J*, (**b**) *FRR*, and (**c**) *R* for DM filtration of O/W emulsions with different feed velocities (DM material: SiO_2_; *TMP*: 0.25 bar; preparation time: 20 min; backwashing pressure: 2 bar).

The flow velocity in cross-flow filtration can affect the deposition of foulants on the DM’s surface. High flow velocity can help prevent concentration polarization, but excessively high flow velocity may damage the DM layer, reducing its ability to mitigate fouling. To investigate the effect of flow velocity on the fouling mitigation ability of the DM, experiments were conducted by preparing the DM for 20 min at a flow velocity of 0.34 m/s and filtering the O/W emulsion at 0.25 bar under different flow velocities. Backwashing was performed at 2 bar. The results are shown in Figure 11.

Figure 11a shows the *J*s of the DM-filtered O/W emulsion at various flow velocities. The *J*_i_s are nearly identical for all feed velocities, while the *J*_s_ increases with the increasing flow velocity up to 0.34 m/s. This is because higher flow velocities reduce the deposition of oil droplets on the DM’s surface [44]. However, when the flow velocity reaches 0.42 m/s, the *J*_s_ decreases because the DM layer is partially carried away by the flow at this high velocity and becomes thinner. Therefore, the ability of the DM to block oil droplets is weakened and the fouling mitigation effect is diminished. The same trend is also observed in *FRR* changes, although the *FRR* values vary only slightly (Figure 11b). There is no significant change in *R* values at different flow velocities (Figure 11c), indicating that flow velocity has little effect on *R* under certain conditions.

#### 3.4.3. Effect of *TMP*

**Figure 12:**
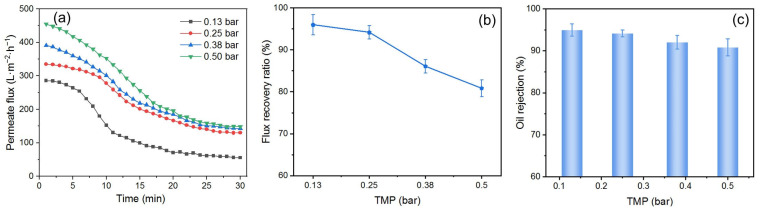
(**a**) *J*, (**b**) *FRR*, and (**c**) *R* for DM filtration of O/W emulsions with different *TMP*s (DM material: SiO_2_; preparation time: 20 min; flow velocity: 0.34 m/s; backwashing pressure: 2 bar).

The *TMP* in cross-flow filtration is also an important parameter in both processes of DM preparation and O/W emulsion filtration. To study the effect of *TMP*, the SiO_2_ DM was prepared for 20 min and used to filter O/W emulsions at different *TMP*s, with a flow velocity of 0.34 m/s and a backwashing pressure of 2 bar for 10 s. The results are shown in Figure 12.

Figure 12a illustrates the *J*s during DM filtration of O/W emulsions at different *TMP*s. The *J*_i_ increases as the *TMP* is increased. A substantial increase in *J*_s_ was observed when the *TMP* was increased from 0.13 to 0.25 bar. However, further increasing the *TMP* from 0.25 to 0.50 bar results in minimal changes to the *J*_s_. This indicates the presence of a critical pressure (*p*_c_) in cross-flow filtration. When the *TMP* is below this *p*_c_, it has a significant effect on the *J*, a phase referred to as the pressure control zone; when the *TMP* exceeds the *p*_c_, its role in regulating *J* diminishes, leading to what is known as the mass transfer control zone. The same phenomenon appears in Buetehorn’s report [52]. The *p*_c_ in this experiment was estimated to be 0.25 bar. The variation patterns of the *FRR* (Figure 12b) and *R* (Figure 12c) as a function of *TMP* are similar to those observed in dead-end filtration. This behavior can be attributed to the deformation of oil droplets under high *TMP*, which allows them to pass through the DM layer and foul the SiC PM.

### 3.5. Comparison of Filtration Modes

**Figure 13:**
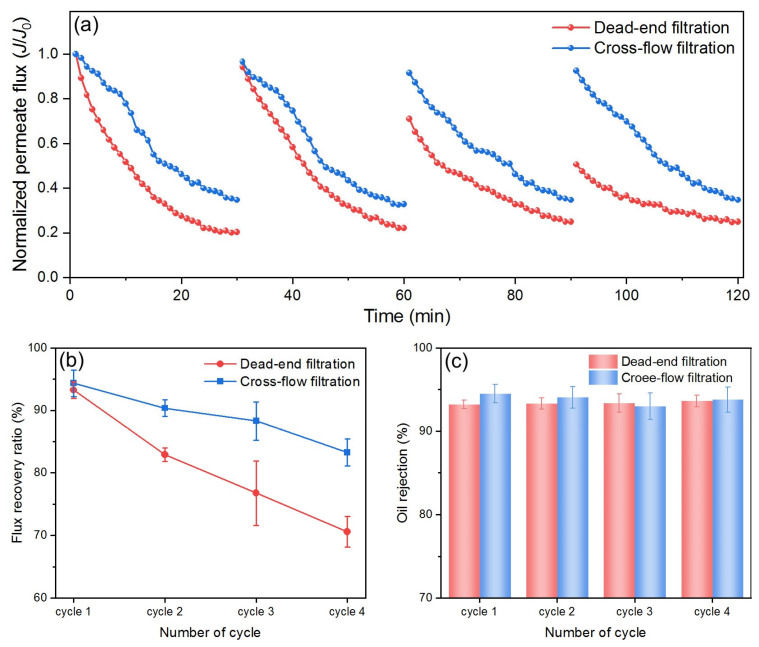
(**a**) Normalized permeate flux, (**b**) *R*, and (**c**) *FRR* for DM filtration of O/W emulsions in different filtration modes.

Cross-flow filtration helps minimize foulant buildup on the membrane surface through tangential flowing, while dead-end filtration tends to rapidly form a cake layer, leading to a quick decline in flux. By comparing DM performance in these two filtration modes, the effects of selected operational parameters on membrane fouling can be better understood, allowing for the optimization of filtration processes. For this purpose, the DM filtration of O/W emulsions was studied under two different filtration modes using optimum parameters from previous sections. Because the optimal DM deposition differs for two modes, the *J*s in different modes cannot be used directly for comparison. Therefore, the fluxes are normalized by dividing all values by their respective *J*_0_ and then compared, as shown in Figure 13a.

Both modes show a decrease in flux with time, which is typical due to oil fouling and cake buildup on the DM’s surface. However, for dead-end mode, the flux decreases more rapidly and reaches a much lower value by the end of each cycle (~0.2), while for cross-flow mode the flux decreases at a slower rate and remains higher (~0.4–0.5 at the end of each cycle). In dead-end filtration, mass transfer is primarily governed by the buildup of a cake layer of oil droplets directly on the DM’s surface, which acts as an additional resistance to flow. The structure and porosity of this cake layer evolve dynamically, leading to a progressive reduction in flux. Furthermore, the cake layer can amplify concentration polarization effects, thereby further decreasing performance. In contrast, cross-flow filtration introduces a shear force parallel to the DM’s surface, which removes a portion of the accumulated foulants and mitigates cake growth. The mass transfer coefficient in cross-flow systems can be estimated using the film theory and appropriate Sherwood correlations, reflecting the enhanced back-transport of solutes and colloids away from the membrane. This results in a slower rate of flux decline, as observed in our experimental results.

After each cycle, the flux in cross-flow mode is restored to near the initial value compared to a significant drop of flux after two cycles in dead-end mode. Throughout the filtration period, cross-flow mode maintains significantly higher flux than dead-end mode. All of these findings demonstrate that cross-flow mode consistently outperforms dead-end mode in all filtration cycles during the O/W emulsion filtration, which is also reflected in the *FRR* comparison, as shown in Figure 13b. In each cycle, the *FRR* for cross-flow filtration is consistently higher than that for dead-end filtration, and it declines more slowly over an increased number of cycles, highlighting the advantages of cross-flow filtration. In terms of *R* values, there is no significant difference between the two filtration modes, both maintaining a value around 94% (Figure 13c). Overall, based on the *J* profiles, the *FRR* values, and the *R* values during the O/W emulsion filtration in the two modes, cross-flow mode proves to be more efficient and suitable for O/W separation applications.

### 3.6. Preliminary Cost Discussion

Effective mitigation of membrane fouling is essential to maximize the economic viability of membranes, especially for SiC membranes, due to its higher prices. SiO_2_-based DMs have the potential to be a cost-effective and sustainable strategy to mitigate fouling of SiC membranes. Firstly, in terms of material cost, SiO_2_ is a widely available and low-cost material, with bulk industrial prices typically ranging from USD 0.5 to USD 1 per kg. Based on our optimized DM deposition amount of 300 g/m^2^, the material cost for SiO_2_ per square meter of membrane is estimated at USD 0.15–0.30. This represents a very minor fraction of the total cost of the SiC membranes, which are generally 4–5 times more expensive than polymeric membranes. Therefore, the incremental cost of the SiO_2_ DM is negligible compared to the value of the SiC membranes it protects. Secondly, in terms of energy consumption, the energy consumed for DM formation and removal (brief stirring and backwashing at 2 bar) is modest compared to the total energy required for the filtration process itself. The increased *FRR* and reduced fouling achieved by the SiO_2_ DM also contribute to lower overall energy consumption per unit of water treated, as higher sustained fluxes reduce the need for frequent cleaning and downtime. Thirdly, in terms of operational savings, without DM protection, SiC membranes suffer rapid flux decline and diminished flux recovery, necessitating frequent chemical cleaning and more frequent replacement. Our results show that the SiO_2_ DM significantly improves flux stability and *FRR*, effectively extending the operational lifespan of SiC membranes. Even a modest increase in membrane lifespan translates to substantial cost savings given the high replacement costs of SiC membranes. Moreover, reduced cleaning frequency minimizes chemical use and related costs, further enhancing economic benefits.

In summary, the application of the SiO_2_-based DM adds only a minor incremental material and energy cost while delivering substantial savings by reducing fouling, lowering cleaning frequency, and extending the lifespan of expensive SiC membranes. Therefore, the SiO_2_-based DM can be a cost-effective and sustainable strategy to mitigate fouling of SiC membranes.

## 4. Conclusions

This study explores the application of a pre-deposited DM as a strategy to mitigate the fouling of SiC membranes during the separation of O/W emulsions. Five oxides (Fe_2_O_3_, SiO_2_, TiO_2_, ZrO_2_, and Al_2_O_3_) were evaluated for their suitability as DM materials. Among these, SiO_2_ emerged as the most effective DM material due to its favorable combination of particle size, negative surface charge (zeta potential: −26.70 mV at pH 5.6), hydrophilicity, and underwater oleophobicity (*OCA* = 138.7°). These properties enabled the SiO_2_ DM to minimize fouling via electrostatic repulsion of oil droplets, achieving high *FRR*s and stable *R*s of ~93% in dead-end mode. Operational parameters were optimized for both dead-end and cross-flow filtration modes. In dead-end mode, a DM deposition amount of 300 g/m^2^, a *TMP* of 0.25 bar, and a backwashing pressure of 2 bar were identified as ideal, balancing flux, fouling resistance, and DM regeneration. Cross-flow mode demonstrated superior performance, maintaining higher normalized permeate flux (~0.4–0.5 vs. ~0.2 in dead-end mode) and slower *FRR* decline over multiple cycles. This was attributed to reduced concentration polarization and efficient DM stabilization under tangential flow. A preparation time of 20 min, a critical *TMP* of 0.25 bar, and a flow velocity of 0.34 m/s were optimal for cross-flow operations, ensuring minimal irreversible fouling while sustaining high separation efficiency. These findings highlight the potential of SiO_2_-based DMs as a cost-effective and sustainable strategy to mitigate fouling of SiC membranes for industrial O/W emulsion separation. Future work could explore long-term DM stability, scalability in real-world conditions, and the integration of hybrid DM materials for enhanced performance.

It is important to note that the O/W emulsion used in this study, comprising vegetable oil and Tween 80, serves as a simplified model to systematically investigate membrane fouling and separation performance. However, real O/W emulsions, such as produced water, are generally more complex, containing various dissolved salts, naturally occurring surfactants, and suspended solids, all of which may influence DM formation, fouling behavior, and overall separation efficiency. As such, caution should be exercised when generalizing these findings to real O/W emulsion systems. Further studies using actual O/W emulsion samples are warranted to comprehensively evaluate the applicability and robustness of DMs under realistic conditions.

## Data Availability

The original contributions presented in this study are included in the article/Supplementary Material. Further inquiries can be directed to the corresponding author(s).

## Supplementary Materials

The following supporting information can be downloaded at https://www.mdpi.com/article/10.3390/membranes15070195/s1, Figure S1: (a) Size distribution of different oxide particles and the droplets of the O/W emulsion. (b) Zeta potentials of five oxides at different pHs; Figure S2: SEM images of the surface of (a) pristine SiC PM and (b) Fe_2_O_3_ DM (deposition amount: 300 g/m^2^); Table S1: Physical properties of DM oxide materials and formed DMs as well as the DM’s filtration performance.
