# Supplementary material for: Fouling Mitigation of Silicon Carbide Membranes by Pre-Deposited Dynamic Membranes for the Separation of Oil-in-Water Emulsions
Source: Membranes (Basel). 2025 Jun 30;15(7):195. doi: 10.3390/membranes15070195 (PMC12299714; doi:10.3390/membranes15070195)
Supplement: Supplementary file 1 [file membranes-15-00195-s001.zip › membranes-3678693-supplementary.pdf]

Fouling Mitigation of Silicon Carbide Membranes by Pre-deposited Dynamic Membranes for the Separation of Oil-in-water Emulsions

Xin Wu[1, 2], Minfeng Fang[1, 2, 3, *], Guanghui Li[1, 2]

1 Innovation Centre for Environment and Resources, School of Chemistry and Chemical Engineering, Shanghai University of Engineering Science, 333 Longteng Road, Shanghai 201620, China

2 China Petroleum and Chemical Industry Key Laboratory of Silicon Carbide Ceramic Membrane, Shanghai University of Engineering Science, 333 Longteng Road, Shanghai 201620, China

3 Zhejiang Motonghuihai Sci & Tech Development Co., Ltd., 1558 Dongpo Road, Huzhou 313000, China.

*Corresponding author. E-mail address: fmf@sues.edu.cn

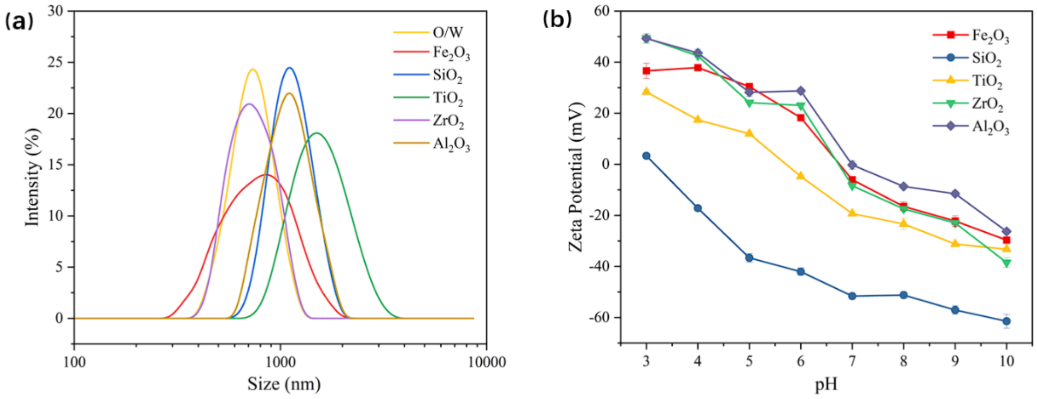

Figure S1 (a) Size distribution of different oxide particles and the droplets of the O/W emulsion. (b) Zeta potentials of five oxides at different pHs.

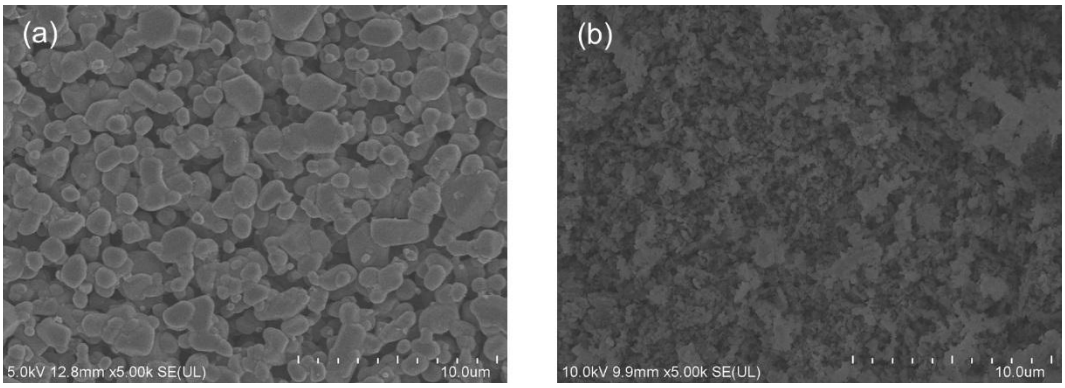

Figure S2 SEM images of the surface of (a) pristine SiC PM and (b) Fe_2O_3 DM (deposition amount: 300 g/m^2).

Table S1 Physical properties of DM oxide materials and formed DMs as well as the DM's filtration performance.

DM material	Isoelectric point at pH 5.6	Zeta potential (mV)	Average particle size (nm)	Surface roughness of DM (μm)	Olephobicity of DM (°)	*FRR* in 1st cycle	*R* in 1st cycle
Fe_2O_3	6.7	23.6	830 ± 50	1.725	124.6	74%	94%
SiO_2	3.2	-40.3	1200 ± 110	0.542	138.7	93%	93%
TiO_2	5.7	2.10	1600 ± 110	0.791	117.6	89%	91%
ZrO_2	6.8	24.4	740 ± 60	1.787	146.3	77%	95%
Al_2O_3	7.0	29.8	1100.0 ± 100	2.047	124.1	82%	93%
